# A possible new syndrome with double endocrine tumors in association with an unprecedented type of familial heart-hand syndrome: a case report

**DOI:** 10.1186/1752-1947-4-347

**Published:** 2010-10-29

**Authors:** Masashi Demura, Takashi Yoneda, Shigehiro Karashima, Toshinori Higashikata, Hiroshi Mabuchi, Mitsuhiro Kawano, Masakazu Yamagishi, Yoshiyu Takeda

**Affiliations:** 1 Division of Endocrinology and Hypertension, Department of Internal Medicine, Graduate School of Medical Science, Kanazawa Universit, 13-1 Takara-machi, Kanazawa, 920-8641, Japan; 2 Department of Internal Medicine, Komatsu Municipal Hospital, HO-60 Mukai Moto-ori-machi, Komatsu, Japan; 3 Department of Lipidology, Graduate School of Medical Science, Kanazawa University Graduate School of Medical Science, Kanazawa University, 13-1 Takara-machi, Kanazawa, 920-8641, Japan; 4 Division of Rheumatology, Department of Internal Medicine, Graduate School of Medical Science, Kanazawa University, 13-1 Takara-machi, Kanazawa, 920-8641, Japan; 5 Division of Cardiology, Department of Internal Medicine, Graduate School of Medical Science, Kanazawa University, 13-1 Takara-machi, Kanazawa, 920-8641, Japan

## Abstract

**Introduction:**

The combination of a pituitary prolactinoma and an aldosterone-producing adrenal adenoma is extremely rare. To the best of our knowledge, double endocrine tumors in association with heart-hand syndrome have not previously been reported.

**Case presentation:**

A 21-year-old Japanese woman presented with galactorrhea and decreased visual acuity. A large pituitary adenoma with an increased level of serum prolactin was apparent by computed tomography. She additionally showed mild hypertension (136/90 mmHg) accompanied by hypokalemia. The plasma aldosterone concentration was increased. Computed tomography showed a mass in the right adrenal gland. No other tumors were found despite extensive imaging studies. Physical and radiographic examinations showed skeletal malformations of the hands and feet, including hypoplasia of the first digit in all four limbs. An atrial septal defect was demonstrated by echocardiography. Similar digital and cardiac abnormalities were detected in our patient's father, and a clinical diagnosis of hereditary heart-hand syndrome was made.

**Conclusion:**

No established heart-hand syndrome was wholly compatible with the family's phenotype. Her father had no obvious endocrine tumors, implying that the parent of transmission determined variable phenotypic expression of the disease: heart-hand syndrome with multiple endocrine tumors from the paternal transmission or no endocrine tumor from the maternal transmission. This suggests that the gene or genes responsible for the disease may be under tissue-specific imprinting control.

## Introduction

The multiple endocrine neoplasia (MEN) syndrome is characterized by the occurrence of tumors involving two or more endocrine glands within a single patient. There are two major forms of MEN, type 1 and 2. MEN1 is characterized by the combined occurrence of tumors of the parathyroids, pancreatic islet cells, and anterior pituitary. MEN2 describes the association of medullary thyroid carcinoma, pheochromocytomas, and parathyroid tumors. MEN1 is caused by mutation in the *MEN1 *gene, while MEN2 is caused by mutation in the *RET *gene [1].

Heart-hand syndromes are a broad category of diseases [2]. The most common form is Holt-Oram syndrome (HOS; MIM No. 142900) caused by a loss-of-function mutation in the *TBX5 *gene located on chromosome 12q24.1 (heart-hand syndrome I) [3]. Diagnosis is based on skeletal abnormalities with a pre-axial radial ray distribution as well as cardiac malformations that typically include atrial and/or ventricular septal defects and arterioventricular nodal dysfunction. Skeletal malformations are limited to the upper limbs. Other forms of heart-hand syndrome have been described [4-8].

## Case presentation

**Figure 1:**
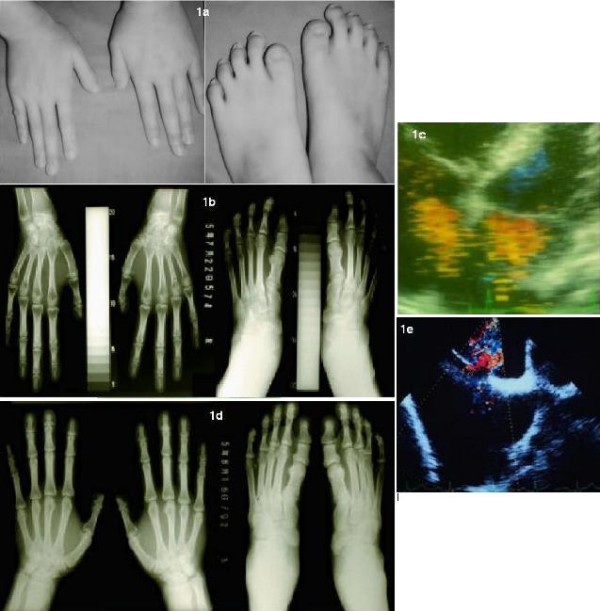
**Patient's phenotype**. **(a) **Photograph of both hands and feet of our patient. **(b) **Roentgenogram of the patient's hands and feet. **(c) **Echocardiogram of the patient **(d) **Roentgenogram of her father's hands and feet. **(e) **Echocardiogram of her father. Note symmetric hypoplasia of the first digit in both our patient and her father. Asterisk (*) displays ASD. RA, right atrium. LA, light atrium.

A 21-year-old Japanese woman presented to our clinic with galactorrhea and decreased visual acuity in the left eye. She was a full-term baby and was delivered after an uncomplicated pregnancy. A heart murmur was detected at age 17. She is 165 cm tall and weighs 52 kg. Physical and radiographic examinations showed skeletal malformations of the hands and feet, including hypoplasia of the first digit in all four limbs (Figure 1a,b). Symmetric phalangeal hypoplasia was observed. An atrial septal defect was demonstrated by echocardiography, with no electrocardiographic evidence of a conduction disturbance (Figure 1c). No additional clinical or radiologic abnormalities were present. Similar digital and cardiac abnormalities were detected in our patient's father (Figure 1d,e), and a clinical diagnosis of hereditary heart-hand syndrome was made. However, no established heart-hand syndrome was wholly compatible with our patient's phenotype.

The serum prolactin concentration was increased (1751 ng/mL; normal, 1.4 to 14.6), and a large pituitary adenoma was apparent on computed tomography (CT). Our patient underwent trans-sphenoidal surgery, which accomplished removal of about 50 percent of this prolactin-secreting tumor (prolactinoma). However, galactorrhea and hyperprolactinemia (200 ng/mL) persisted. Administration of bromocriptine ameliorated these abnormalities.

Our patient additionally showed stage 1 hypertension (JNC 7, 136/90 mmHg) accompanied by hypokalemia (serum potassium, 2.6 mEq/L). The plasma aldosterone concentration was increased (738.4 pg/mL; normal, 20.0 to 130.0), and plasma renin concentration was decreased (less than 2.0 pg/mL; normal, 2.5 to 21.4). CT disclosed a mass in the right adrenal gland. The tumor was resected and diagnosed histologically as adrenocortical adenoma. Hypertension, hypokalemia, and hyporeninemic hyperaldosteronemia all resolved upon resection, and a diagnosis of aldosterone-producing adenoma was made. No other tumors were found despite extensive imaging studies. The combination of prolactinoma and aldosterone-producing adenoma does not correspond to any known multiple-endocrine-tumor syndrome.

Except with respect to lower-limb abnormalities, the individuals here described closely resembled the HOS phenotype. Considering the possibility of contiguous gene syndrome, mutational analysis concerning the *TBX5 *gene was performed in our patient. By metaphase chromosomal analysis her karyotype was 46,XX (normal female) and G-banding of the prometaphase chromosome 12 in peripheral lymphocytes was also normal. We next analyzed transcripts of the *TBX5 *gene in lymphoblast cells (LBC). Only a novel splice-variant of the *TBX5 *mRNA omitting exon 2 (DDBJ Accession No. AB051068) was expressed in both our patient and normal subjects; direct sequencing of *TBX5 *cDNA from our patient's LBC disclosed no mutation. Complete gene deletion was unlikely because of absence of allelic loss on 12q24.1 as determined using a polymorphic marker of intron 2 (D12S1646). These various findings argued strongly against a mutation of the *TBX5 *gene.

Despite no report on identified *MEN1 *mutations in patients with a prolactinoma and an aldosterone-producing adenoma, the rare combination of tumors might occur in the MEN1 patients. Mutational analysis, however, showed no mutation in the *MEN1 *gene. She possessed two alleles of polymorphic markers of PYGM and D11S4946, showing no loss of heterozygosity on the MEN1 locus.

## Conclusion

We report the case of a female patient with double endocrine tumors (prolactinoma and aldosterone-producing adenoma) in association with familial heart-hand syndrome. A combination of prolactinoma and aldosterone-producing adenoma was rare [9]. We found no mutation in the *TBX5 *and *MEN1 *genes. These findings suggested that another genetic factor was involved in the complex of double endocrine tumors, atrial septal defect and pre-axial brachydactyly in this family.

Recent studies have indicated that Tbx5 is important for normal development of the upper limb in the chick, while Tbx4 is important for the lower limb [10]. Upstream regulatory genes for *TBX4 *(on chromosome 16) and for *TBX5 *may be involved in the present case.

**Figure 2:**
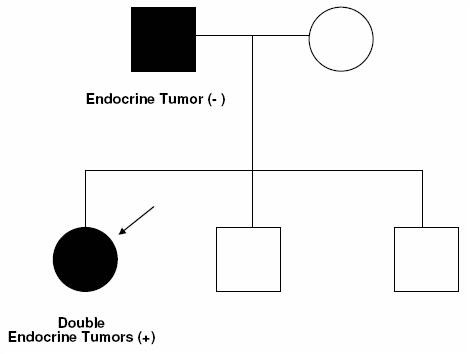
**Pedigree of the family**. Squares represent males, circles represent females, closed symbols indicate affected status, open symbols indicate unaffected status, an arrowhead indicates the proband.

Prolactinoma and aldosterone-producing adenoma, an extremely rare combination of endocrine tumors, were present in the proband but not in the father (Figure 2). Many genes are imprinted in a tissue-specific manner, with monoallelic expression in some cell types and biallelic expression in others. Paternal inheritance of a mutation at some imprinted locus could lead to heart-hand syndrome plus endocrine tumors while maternal inheritance of the same mutation could lead to heart-hand syndrome without endocrine tumors.

Since inheritance of these tumors is not obvious in our proband, she may exhibit multiple genetic disorders. We, however, suspect that she had a germ-line mutation of an unknown gene associated with endocrine tumorigenesis under tissue-specific imprinting control. We speculate that a close association might exist in some other patients between a novel type of heart-hand syndrome and rare combinations of endocrine tumors.

## Consent

Written informed consent was obtained from the patient and her father for publication of this case report and any accompanying images. A copy of the written consent is available for review by the journal's Editor-in-Chief.

## Competing interests

The authors declare that they have no competing interests.

## Authors' contributions

MD had primary responsibility for drafting the manuscript. TY, TH, HM, and YT contributed to patient's evaluation. MD, SK, and MK performed genetic testing for HOS, and MEN1. TY, TH, HM, MY, and YT were involved in the patient's clinical assessment and treatment. All authors read and approved the final manuscript.
